# Intracellular Networks of the PI3K/AKT and MAPK Pathways for Regulating *Toxoplasma gondii*-Induced IL-23 and IL-12 Production in Human THP-1 Cells

**DOI:** 10.1371/journal.pone.0141550

**Published:** 2015-11-03

**Authors:** Juan-Hua Quan, Jia-Qi Chu, Jaeyul Kwon, In-Wook Choi, Hassan Ahmed Hassan Ahmed Ismail, Wei Zhou, Guang-Ho Cha, Yu Zhou, Jae-Min Yuk, Eun-Kyeong Jo, Young-Ha Lee

**Affiliations:** 1 Department of Gastroenterology, The Affiliated Hospital of Guangdong Medical College, Zhanjiang 524-001, Guangdong, China; 2 Stem Cell Research and Cellular Therapy Center, The Affiliated Hospital of Guangdong Medical College, Zhanjiang, 524-001, China; 3 Department of Medical Education, Chungnam National University School of Medicine, Daejeon, 301-131, Korea; 4 Department of Infection Biology, Chungnam National University School of Medicine, Daejeon, 301-131, Korea; 5 Department of Microbiology and Infection Signaling Network Research Center, Chungnam National University School of Medicine, Daejeon, 301-131, Korea; Duke University Medical Center, UNITED STATES

## Abstract

Interleukin (IL)-23 and IL-12 are closely related in structure, and these cytokines regulate both innate and adaptive immunity. However, the precise signaling networks that regulate the production of each in *Toxoplasma gondii*-infected THP-1 monocytic cells, particularly the PI3K/AKT and MAPK signaling pathways, remain unknown. In the present study, *T*. *gondii* infection upregulated the expression of IL-23 and IL-12 in THP-1 cells, and both cytokines increased with parasite dose. IL-23 secretion was strongly inhibited by TLR2 monoclonal antibody (mAb) treatment in a dose-dependent manner and by TLR2 siRNA transfection, whereas IL-12 secretion was strongly inhibited by TLR4 mAb treatment dose-dependently and by TLR4 siRNA transfection. IL-23 production was dose-dependently inhibited by the PI3K inhibitors LY294002 and wortmannin, whereas IL-12 production increased dose-dependently. THP-1 cells exposed to live *T*. *gondii* tachyzoites underwent rapid p38 MAPK, ERK1/2 and JNK activation. IL-23 production was significantly upregulated by the p38 MAPK inhibitor SB203580 dose-dependently, whereas pretreatment with 10 μM SB203580 significantly downregulated IL-12 production. ERK1/2 inhibition by PD98059 was significantly downregulated IL-23 production but upregulated IL-12 production. JNK inhibition by SP600125 upregulated IL-23 production, but IL-12 production was significantly downregulated dose-dependently. *T*. *gondii* infection resulted in AKT activation, and AKT phosphorylation was inhibited dose-dependently after pretreatment with PI3K inhibitors. In *T*. *gondii*-infected THP-1 cells, ERK1/2 activation was regulated by PI3K; however, the phosphorylation of p38 MAPK and JNK was negatively modulated by the PI3K signaling pathway. Collectively, these results indicate that IL-23 production in *T*. *gondii*-infected THP-1 cells was regulated mainly by TLR2 and then by PI3K and ERK1/2; however, IL-12 production was mainly regulated by TLR4 and then by p38 MAPK and JNK. Our findings provide new insight concerning the intracellular networks of the PI3K/AKT and MAPK signaling cascades for regulating *T*. *gondii*-induced IL-23 and IL-12 secretion in human monocytic cells.

## Introduction

*Toxoplasma gondii* is an obligate intracellular protozoan parasite that infects one-third of the world’s population. Almost 80–90% of primary *T*. *gondii* infections are asymptomatic; however, these infections cause various diseases, including lymphadenitis, congenital infection of fetuses, and life-threatening toxoplasmic encephalitis in immunocompromised individuals [1]. Underscoring the success of *T*. *gondii* is a delicate balance between the host immune response, which tries to clear the parasite, and the immune evasion strategies or immunomodulation elicited by the parasite, which enables the ultimate survival of both the parasite and host [2]. The interleukin-12 (IL-12) cytokine family plays a pivotal role in the initiation and regulation of cell-mediated immunity and comprises IL-12, IL-23 and IL-27 [3]. IL-12 has been widely accepted as an important regulator of T-helper 1 cell (Th1) responses and is mostly produced by activated hematopoietic phagocytic cells (monocytes, macrophages, neutrophils) and dendritic cells [4]. IL-12 is a heterodimeric cytokine of 70 kDa comprising covalently linked p40 and p35 subunits, the genes of which are independently regulated. IL-23 is a recently discovered cytokine that is composed of the p19 and p40 subunit, and the IL-12Rβ1 chain of the IL-12 receptor is shared with IL-23 [5,6]. IL-23 is produced by similar cell types as IL-12, and the receptor complex is expressed or upregulated on T and NK cells, as well as on phagocytic hematopoietic cells and dendritic cells (DCs) [7]. There are many reports concerning IL-12 production after *T*. *gondii* infection; however, reports on *T*. *gondii*-induced IL-23 secretion are limited.

Toll-like receptors (TLRs) are pattern-recognition receptors that recognize pathogen-associated molecular patterns synthesized by microorganisms. All TLRs, with the exception of TLR3, associate with the adaptor protein myeloid differentiation primary-response protein (MyD88). Binding of a ligand to its TLR typically stimulates pro-inflammatory activity, which protects the host from pathogen invasion [8]. The importance of TLRs in innate resistance to *T*. *gondii* was demonstrated by MyD88^-/-^ mice being acutely susceptible as IL-12^-/-^ mice to infection with avirulent strains of the parasite, and both TLR2 and TLR4 receptors may participate in the host defense against *T*. *gondii* infection [9,10]. Thus, signaling through TLRs is clearly important in innate resistance to *T*. *gondii*; however, which TLRs are involved in the production of each cytokine has not been extensively studied.

Phosphoinositide-3 kinases (PI3Ks) are a family of signal transduction enzymes involved in critical cellular functions needed to maintain physiological homeostasis, including cellular growth, proliferation, and survival. This pathway is activated by a combination of ligands such as lipopolysaccharide (LPS), and various cell surface receptors such as TLRs, insulin receptor, estrogen receptor, and numerous cytokine receptors [11]. *T*. *gondii* exploits heterotrimeric Gi-protein-mediated signaling to activate PI3K, leading to phosphorylation of the downstream serine/threonine kinase AKT (also known as protein kinase B) and extracellular signal-regulated protein kinases 1/2 (ERK1/2), and inhibition of apoptosis [12]. The mitogen-activated protein kinase (MAPK) family controls gene expression and immune function, and has roles in the positive and negative regulation of proinflammatory cytokine production [13]. There are three major groups of MAPKs in mammalian cells: p38 MAPK, ERK1/2, and c-Jun N-terminal kinases (JNK), also known as stress-activated protein kinases (SAPK). In macrophages that are infected with *T*. *gondii*, all three MAPK signaling modules are rapidly activated, and phosphorylation returns to basal levels within 2 h post-infection [14]. IL-12 production in macrophages in response to *T*. *gondii* is dependent on the TRAF6-dependent phosphorylation of p38 MAPK and ERK1/2, and expression of JNK2 plays a role in *T*. *gondii*-induced immunopathology [15,16]. Many investigators have revealed the signaling pathways in *T*. *gondii*-infected cells; nevertheless, cytokine-specific intracellular signaling networks after *T*. *gondii* infection are still poorly understood.

*T*. *gondii* is a master manipulator of immunity. After encountering *Toxoplasma* and immune cells, proinflammatory signaling cascades may be dramatically triggered within infected cells leading to immune activation or immune subversion. Macrophages, dendritic cells, or neutrophils infected with *T*. *gondii* secrete several cytokines, including IL-23 and IL-12 [4]. IL-23 has a similar structure as IL-12; however, the functions of these cytokines do not overlap in cells infected with *T*. *gondii*, and there is limited information on the regulation of signaling sequences related to their production in *T*. *gondii*-infected human THP-1 monocytic cells. Therefore, to elucidate and compare the signaling cascades responsible for the production of IL-23 and IL-12 in *T*. *gondii*-infected human monocytic THP-1 cells, we examined the involvement of the TLR2/TLR4, PI3K/AKT, and MAPK signaling pathways after treatment with specific antibodies, specific inhibitors, transfection with siRNA designed to target TLR2 or TLR4 using western blotting, RT-PCR, and ELISA.

## Materials and Methods

### Reagents and antibodies

The primary antibodies against phosphorylated extracellular signal-regulated kinase 1/2 (p-ERK1/2), total ERK1/2 (ERK1/2), phosphorylated c-Jun N-terminal kinase (p-JNK), total JNK (JNK), phosphorylated p38 mitogen-activated protein kinase (p-p38 MAPK), total p38 MAPK (p38 MAPK), phosphospecific AKT (Ser473) and total AKT (AKT) were purchased from Cell Signaling Technology Inc. (Danvers, MA, USA). Antibodies against TLR2 and TLR4 were obtained from eBioscience (San Diego, CA, USA). The ERK1/2 inhibitor PD98059, JNK inhibitor SP600125 and p38 MAPK inhibitor SB203580 were obtained from Calbiochem (San Diego, CA, USA). TLR2 agonist Pam_3_CSK_4_ and TLR4 agonist LPS, and PI3K inhibitors, LY294002 and wortmannin, were obtained from Sigma (St. Louis, MO, USA). Secondary antibodies, anti-rabbit-horseradish peroxidase (HRP) and anti-mouse-HRP were also purchased from Sigma.

### Host cells and *T*. *gondii* maintenance

Tachyzoites of the *T*. *gondii* RH strain were multiplied *in vitro* in human retinal pigment epithelium cells (ARPE-19) (American Type Culture Collection, Manassas, VA, USA) and cultured in a 1:1 mixture of Dulbecco’s Modified Eagle Medium (DMEM) and nutrient mixture F12 (DMEM/F12) containing 10% heat-inactivated fetal bovine serum (FBS) and antibiotic—antimycotic (Gibco-Invitrogen, Carlsbad, CA, USA). ARPE-19 cells were infected with the RH strain of *T*. *gondii* at a multiplicity of infection (MOI) of 5. Six hours after inoculation, the cultures were washed twice with PBS to remove any non-adherent parasites and cultured in a 5% CO_2_ atmosphere at 37°C for 2–3 days. Following spontaneous host cell rupture, the parasite and host-cell debris were washed in cold PBS, and the final pellet was resuspended in cold RPMI and passed through a 26-gauge needle and a 5 μm pore size polycarbonate membrane (Millipore, Bedford, MA, USA) to remove ARPE-19 cells.

### Human monocytic THP-1 cells

THP-1 cells are a promonocytic cell line derived from a patient with acute lymphocytic leukemia (ATCC TIB-202) that produce sufficient IL-12 in a *T*. *gondii* model [17]. THP-1 cells were cultured in RPMI 1640 medium (Gibco-Invitrogen) supplemented with 10% FBS, 2 mM L-glutamine, 25 mM HEPES, and antibiotic—antimycotic (Gibco-Invitrogen). Cell cultures were maintained at 37°C and 5% CO_2_, and cell viability was determined using the Trypan blue dye-exclusion assay.

### RNA extraction and semi-quantitative RT-PCR

Total cellular RNA was extracted from normal THP-1 cells and those infected with *T*. *gondii* using TRIzol reagent (Invitrogen Life Technology, Auckland, New Zealand), and semi-quantitative RT-PCR was performed. mRNA was reverse transcribed to cDNA using Superscript II reverse transcriptase (Invitrogen Life Technology) as described by the manufacturer. The primer sequences for PCR were designed as follows: p19-F (5′-GAG CAG CAA CCC TGA GTC CCT A-3′), p19-R (5′-CAA ATT TCC CTT CCC ATC TAA TAA-3′), p40-F (5′-GCT ATG GTG AGC CGT GAT-3′), p40-R (5′-CAT GCT AAT GAG AAA GGG ATT-3′), p35-F (5′-AGG CCC TGA ATT TCA ACA-3′), p35-R (5′-GAT GTA ATA GTC CCA TCC TTC TTT-3′), HPRT-F (5′-TGT GTG CTC AA GGG GGG C-3′), and HPRT-R (5′-CGT GGG GTC CTT TTC ACC-3′). Amplified products were electrophoresed in 1.5% agarose gels and visualized with ethidium bromide. The band intensities were analyzed densitometrically using Image J software (National Institutes of Health, Bethesda, MD, USA).

### Cytokine ELISA

THP-1 cells were incubated with TLR4 agonist Pam3CSK4 or TLR2 agonist LPS for 18h, or infected for 18 h with the RH strain of *T*. *gondii* at a MOI of 0.1, 1, or 10. Triplicate supernatants of treated or untreated THP-1 cells were collected, and the levels of both IL-23 and IL-12 were measured using commercially available IL-23 and IL-12 ELISA kits according to the manufacturer’s instructions (R&D Systems, Inc., Minneapolis, MN, USA). Cytokine concentrations in the samples were calculated using standard curves generated from recombinant cytokines, and the results were expressed in picograms per milliliter.

### siRNA transfection

Negative-control scrambled small interfering RNA (siRNA), TLR2-targeting siRNA, and TLR4-targeting siRNA were purchased from Santa Cruz Biotechnology (Santa Cruz, CA, USA) and used following the manufacturer’s instructions. THP-1 monocytes were seeded at 1×10^6^ cells per well in a six-well plate. The next day, THP-1 cells were transfected with siRNAs (25 pmol) using Lipofectamine RNAiMAX (Invitrogen) and cultured for 48 h prior to *T*. *gondii* infection.

### Antibody neutralization assay

THP-1 cells (1.0 × 10^6^ cells/mL) were added to a 48-well cell culture plate and pre-treated with 1–10 μg/mL TLR2 and TLR4 monoclonal antibodies, or IgG isotype control for 1 h at 37°C in 5% CO_2_. Antibodies and isotype controls used were functional grade anti-human TLR2, TLR4 and IgG isotype control antibodies from eBioscience. Following the 1 h incubation, *T*. *gondii* tachyzoites infected into THP-1 cells in the continuing presence of neutralizing antibodies and the cells were further incubated for 18 h in the same conditions. IL-23 and IL-12 from cell supernatants were determined as described above.

### Western blot analysis

THP-1 cells were infected in a MOI of 10 for 10, 20, 30, 60, and 120 min with or without preincubation with specific inhibitors or monoclonal antibodies. *T*. *gondii*-infected or uninfected THP-1 cells were washed with PBS, and proteins were extracted using PRO-PREP^™^ Protein Extraction Solution (iNtRON Biotechnology, Seoul, Korea) for 15 min on ice. After centrifugation at 4°C for 15 min at 14,000 *g*, the supernatants were collected, and equal amounts of protein from each sample were separated by standard sodium dodecyl sulfate-polyacrylamide gel electrophoresis (SDS-PAGE) and transferred to polyvinylidene difluoride membranes. For immune detection of specific proteins, the membranes were blocked with 5% skim milk in Tris-buffered saline (20 mM Tris, 137 mM NaCl, pH 7.6) containing 0.1% Tween 20 (TBST). After washing once in TBST, the membranes were incubated overnight at 4°C with anti-phospho AKT (Ser473), anti-phospho AKT (Thr308), anti-AKT, anti-phospho p38 MAPK, anti-phospho ERK1/2, anti-phospho JNK, anti-p38 MAPK, anti-ERK1/2, and anti-JNK (all from Cell Signaling Technology, Beverly, MA, USA), diluted in 5% bovine serum albumin in TBST. Following three washes in TBST, the membranes were incubated for 120 min with horseradish peroxidase-conjugated anti-mouse or anti-rabbit IgG (Jackson Immuno Research Laboratories, West Grove, PA, USA) diluted at 1:10,000 in incubation buffer as above. After extensive washes, the bound antibodies were visualized by ECL chemiluminescence (Amersham Biosciences, Freiburg, Germany).

### Statistical analyses

Results are expressed as means ± standard deviations (SDs) of at least three independent experiments unless otherwise indicated. The statistical significance of the data was determined by unpaired Student’s *t*-test. *P* values less than 0.05 were deemed to be statistically significant.

## Results

### Study design

In order to find out the signaling cascades responsible for the expression of IL-23 and IL-12, we examined the involvement of the TLR2/TLR4, PI3K, AKT, and MAPK signaling pathways in *T*. *gondii*-infected THP-1 cells by ELISA or western blotting analysis. The specific role of these signaling pathways were intervened by treatment with monoclonal antibodies (mAb) and/or specific inhibitors or agonists or transfection with siRNA designed to target TLR2 or TLR4. We first examined the expression of IL-23 and IL-12 in *T*. *gondii*-infected human THP-1 monocytic cells by RT-PCR and ELISA. Next, to evaluate which TLRs are responsible for the production of IL-23 or IL-12 in *T*. *gondii*-infected THP-1 cells, the cells were incubated with anti-TLR2 or anti-TLR4 mAb or transfected with siRNAs against TLR2, TLR4, control siRNA or treated with TLR4 or TLR2 agonist, and then infected with live *T*. *gondii* tachyzoites for 18 h; subsequently, IL-23 and IL-12 levels from the culture supernatants were measured by ELISA. To examine the involvement of PI3K/AKT and MAPK signaling pathways in the production of IL-23 and IL-12, THP-1 cells were pretreated with specific PI3K inhibitors (LY294002 and wortmannin), p38 MAPK inhibitor (SB203580), JNK inhibitor (SP600125), and ERK1/2 inhibitor (PD98059), and then infected with *T*. *gondii*, followed by detection of the phosphorylation levels by western blotting using anti-p-AKT, anti-p-p38 MAPK, anti-p-ERK1/2, or anti-p-JNK.

### *T*. *gondii* infection upregulates the expression of IL-23 and IL-12

**Fig 1:**
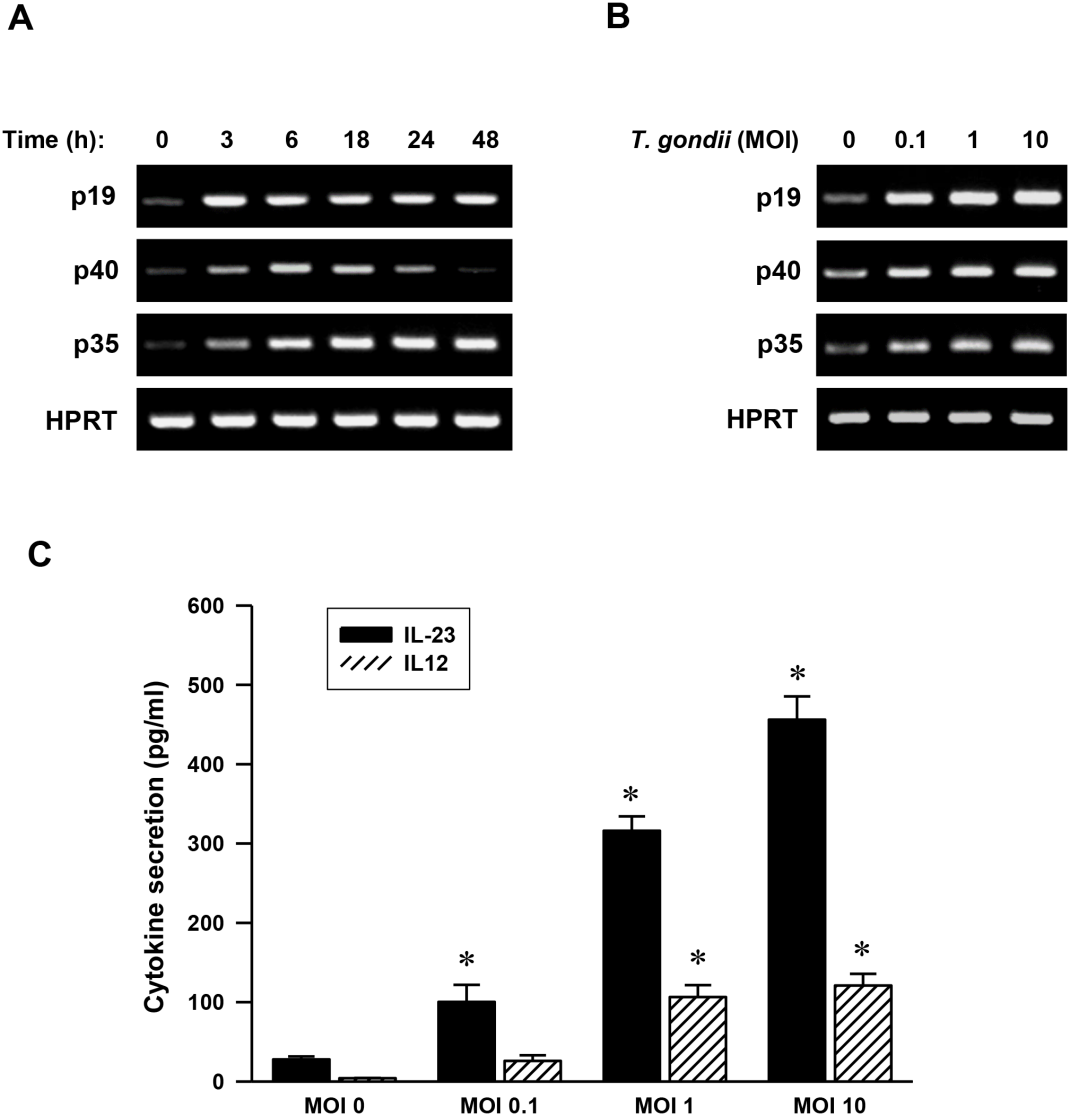
*Toxoplasma gondii* infection induces IL-23 and IL-12 expression in human monocytic THP-1 cells. (A) Kinetics of *T*. *gondii* induction of p19, p40, and p35 mRNA expression. Human monocytic THP-1 cells were treated with *T*. *gondii* at a multiplicity of infection 1 (MOI 1) for the times indicated. RNA was extracted, and semi-quantitative RT-PCR for p19, p40, and p35 was performed. HPRT was used as a loading control. A representative gel of three independent replicates with similar results was shown. (B) MOI-dependent increases in the mRNA expression of p19, p40, and p35. RT-PCR analysis for p19, p40, and p35 mRNA expression at 6 h was assessed in THP-1 cells after treatment with *T*. *gondii* at a MOI of 0, 0.1, 1, or 10. (C) MOI-dependent increases in IL-23 and IL-12 production. ELISA for IL-23 and IL-12 was performed in THP-1 cells after treatment with *T*. *gondii* at a MOI of 0, 0.1, 1, or 10 for 18 h. The results, expressed as means ± SDs of values for triplicate wells, are representative of three separate experiments.* *P*<0.05 compared with uninfected group (MOI 0).

IL-12 and IL-23, two heterodimeric cytokines mostly produced by activated hematopoietic phagocytic cells (monocytes, macrophages, neutrophils) and dendritic cells, are composed of a specific polypeptide, namely p35 and p40 for IL-12 and p19 and p40 for IL-23 [4]. To assess how the stimulation of THP-1 cells with *T*. *gondii* regulates the expression of IL-23 (p19 and p40 subunits) and IL-12 (p35 and p40 subunits), key inflammatory cytokines in immune responses, we analyzed the kinetics of IL-23 and IL-12 expression starting from 3 h up to 48 h by adding fresh, live *T*. *gondii* tachyzoites at a MOI of 1. The mRNA expression patterns of p19 and p35 were similar. They started be markedly induced from 3 h and their level remained high up to 48 h post-infection. However, the p40 mRNA level peaked at 6 h post-infection and gradually decreased (Fig 1A). Thus, we selected *T*. *gondii* induced p19, p40 and p35 expression of time point 6 h and checked these mRNA levels after infected with different MOIs of *T*. *gondii*. p19, p40 and p35 mRNA increments were detected in the *T*. *gondii* infected THP-1 cells. However, there was no significant difference among the groups infected with different MOIs of the *T*. *gondii* (Fig 1B). These data demonstrate that THP-1 cells infected with live *T*. *gondii*, highly express mRNAs coding for IL-12 (p40 and p35 subunits) and IL-23 (p40 and p19 subunits) with a *T*. *gondii* dose-dependent manner.

In a similar manner to changes in the IL-23 and IL-12 mRNA levels, the secretion of IL-23 and IL-12 cytokines also increased with parasite dose. The secretion of IL-23 and IL-12 in *T*. *gondii*-infected THP-1 cells at a MOI of 0.1, 1, and 10 were significantly higher than those of uninfected cells (*P*<0.05). In addition, the secretion levels of IL-23 at the same MOI as that of parasite-infected cells were significantly higher than those of IL-12 (*P*<0.05). At *T*. *gondii* MOI 10, the concentration of IL-23 secretion was about four-fold higher than that of IL-12. IL-23 secretion continuously increased even after MOI 0.1, whereas IL-12 secretion showed a significant increase from MOI 1 (Fig 1C). To evaluate the viability of *T*. *gondii*-infected THP-1 cells, the percentage of the live THP-1 cells treated with *T*. *gondii* at a MOI of 0, 0.1, 1, or 10 for 18 h were assayed by using CellTiter 96^®^AQueous One Solution Cell Proliferation Assay Kit (Promega, Madison, WI). There was no significant difference of percentage of the live THP-1 cells treated with MOI 0.1, MOI 1 and MOI 10 for 18 h as compared with control MOI 0 group (S1 Fig). These results indicate that IL-23 and IL-12 were produced in THP-1 cells by *T*. *gondii* infection in a parasite dose-dependent manner, and also the level of induction of IL-23 was much larger than that of IL-12.

### *T*. *gondii*-induced expression of IL-23 is mainly dependent on TLR2, whereas IL-12 is mainly dependent on TLR4

**Fig 2:**
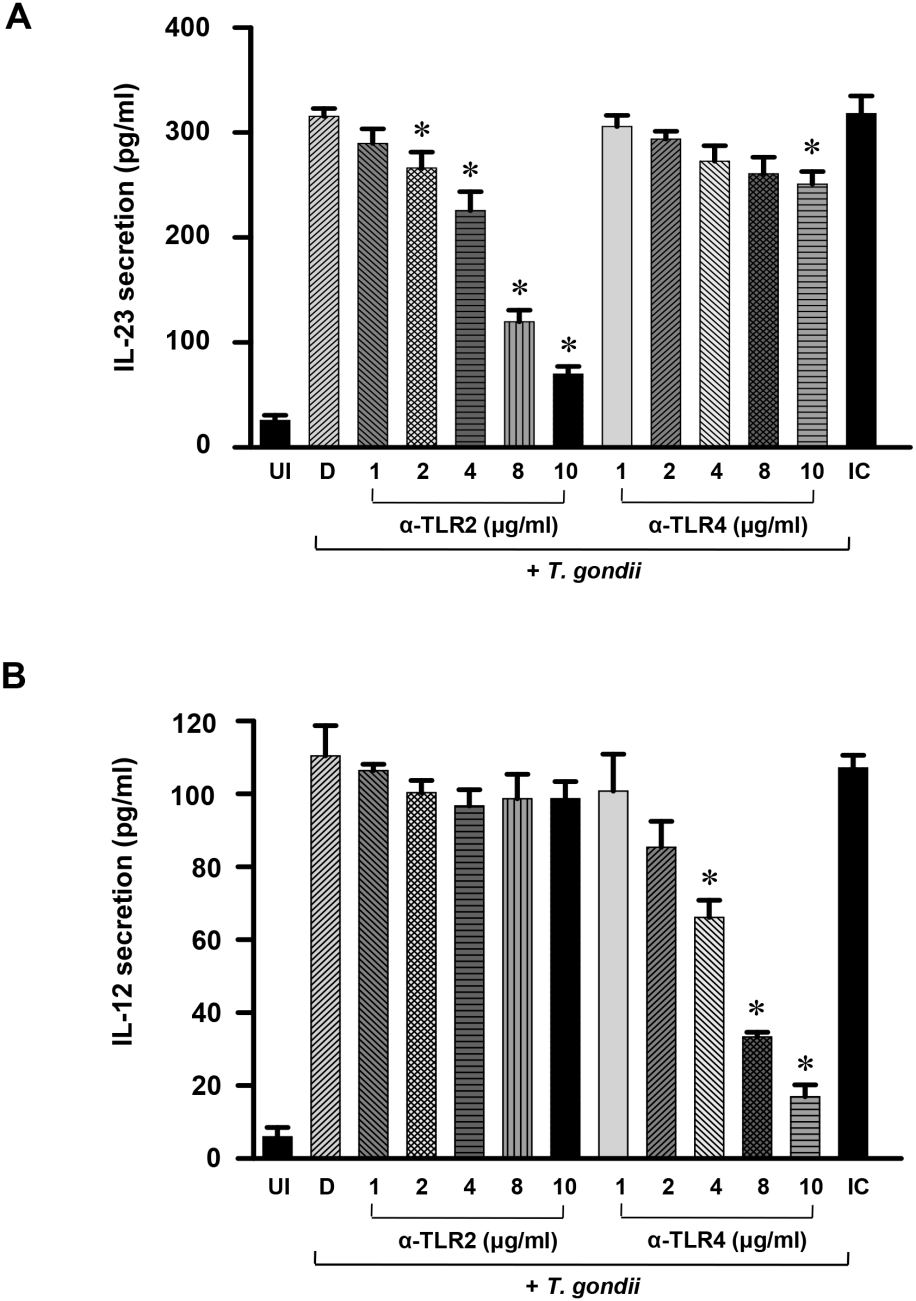
Differential regulation of IL-23 and IL-12 productions in *T*. *gondii*-infected THP-1 cells by pretreatments of anti-TLR2 and anti-TLR4 monoclonal antibodies. THP-1 cells were preincubated with anti-TLR2, anti-TLR4 or isotype-matched control (IC) (10 μg/mL) and infected with *T*. *gondii* sat a MOI 1 subsequently for 18 h. The culture supernatants were harvested and IL-23 (A) and IL-12 (B) productions were measured by ELISA. UI, uninfected control; D, DMSO (0.1%) control; IC, infected control. One representative experiment performed in triplicate is shown.* *P*<0.05 compared with DMSO control (D).

Both the TLR2 and TLR4 receptors may participate in host defense against *T*. *gondii* infection [10]. To examine whether these TLRs are responsible for the *T*. *gondii*-induced IL-23 and IL-12 cytokine responses, THP-1 cells were preincubated with or without anti-TLR2 or anti-TLR4 mAbs, or isotype-matched control antibody, and the cytokine levels were measured by ELISA. *T*. *gondii*-induced IL-23 secretion was strongly inhibited by anti-TLR2 mAb treatment in a dose-dependent manner (2 μg/mL, 15.6%; 4 μg/mL, 28.4%; 8 μg/mL, 62.0%; 10 μg/mL, 77.9%) (*P*<0.05), whereas it changed only slightly after anti-TLR4 mAb treatment (10 μg/mL, 20.5%) (Fig 2A). In contrast, *T*. *gondii*-induced IL-12 secretion was strongly inhibited by anti-TLR4 mAb treatment in a dose-dependent manner (2 μg/mL, 22.7%; 4 μg/mL, 40.1%; 8 μg/mL, 69.7%; 10 μg/mL, 84.5%) (*P*<0.05), whereas there was no change after pretreatment with anti-TLR2 mAb (Fig 2B).

**Fig 3:**
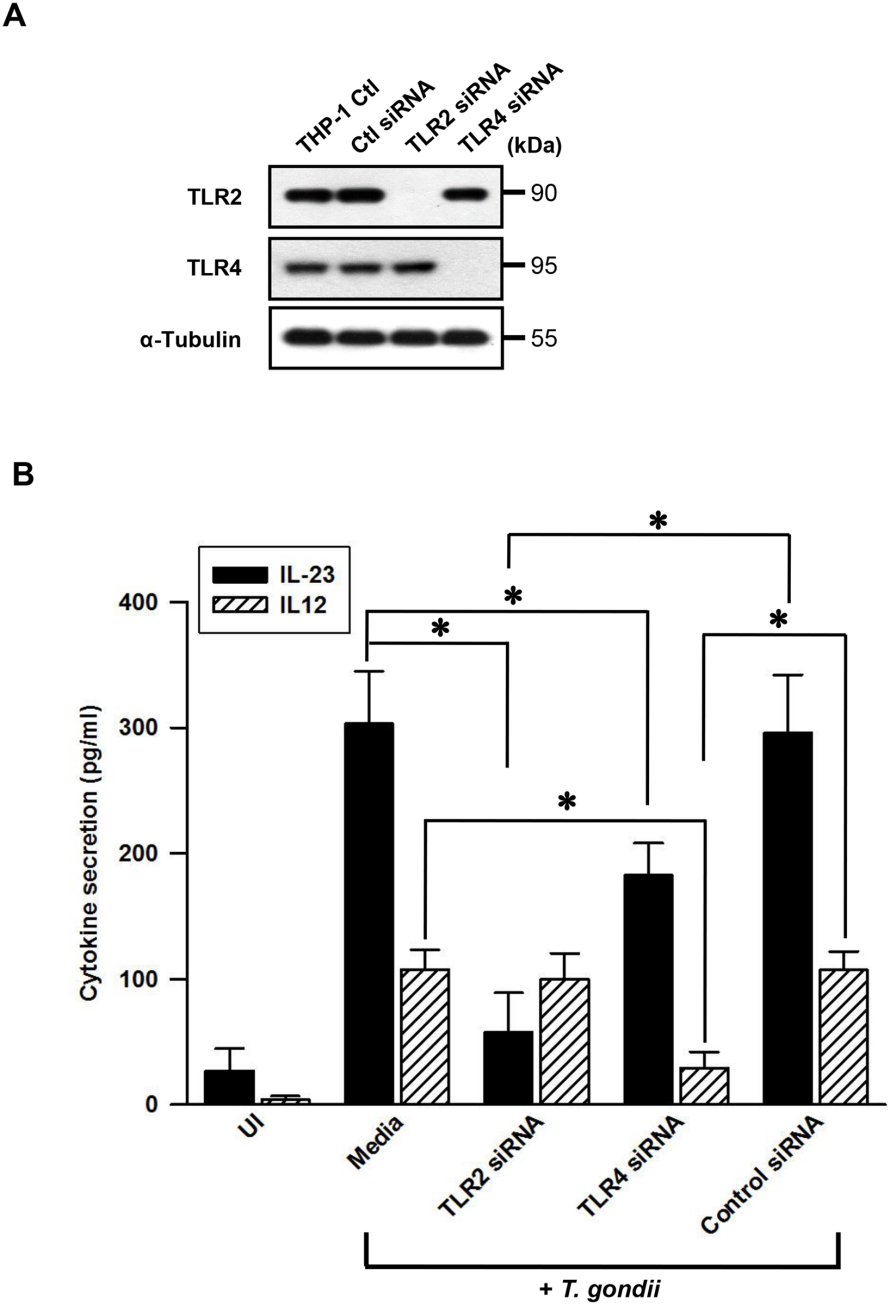
Differential regulation of IL-23 and IL-12 productions in *T*. *gondii*-infected THP-1 cells upon treatment with small interfering RNA (siRNA) of TLR2 or TLR4. (A) THP-1 cells were transfected with 25 pmol of siRNA against TLR2 or TLR4 (or control siRNA) for 48 h, the TLR2 and TLR4 protein expression were determined by western blot analysis. (B) THP-1 cells were treated as Fig 3A, the supernatants were harvested after treatment with *T*. *gondii* at a MOI 1 for 18 h. The secretion levels of IL-23 and IL-12 were determined using ELISA. The results, expressed as means ± SDs of values for triplicate wells, were representative of three separate experiments.* *P*<0.05 compared with medium control or siRNA control.

To confirm the specific effects of each anti-TLR antibody treatment on IL-12 or IL-23 production, THP-1 cells were transfected with siRNAs against TLR2 or TLR4 (or control siRNA). The western blot analysis of target proteins for knockdown efficiency of each siRNA transfection against TLR2 or TLR4 showed a complete decrease of targeted TLR2 or TLR4 protein without affecting protein expression of the other TLR form (Fig 3A). Next, we checked the functional activities of THP-1 cells transfected with siRNAs against TLR2 or TLR4 (or control siRNA). As shown in S1 File, when control siRNAs-transfected THP-1 cells were treated with different concentration of TLR2 agonist Pam_3_CSK_4_, IL-23 production was markedly increased in a dose-dependent manner. Though, Pam_3_CSK_4_-dependent IL-12 production was not increased in a dose-dependent manner (Fig A in S1 File). When TLR2 siRNA-transfected THP-1 cells were stimulated by Pam_3_CSK_4_, IL-12 production was not significantly decreased by transfection of TLR2 siRNA. However, Pam_3_CSK_4_-stimulated IL-23 production was strongly decreased by transfection of TLR2 siRNAs (Fig A in S1 File). These results suggest that IL-23 production is dependent on TLR2. In addition, the small increase of IL-12 production is not really dependent on TLR2. When control siRNAs-transfected THP-1 cells were treated with different concentration of TLR4 agonist LPS, both IL-23 and IL-12 production was markedly increased in a dose-dependent manner (Fig B in S1 File). When THP-1 cells were transfected with TLR4 siRNAs and stimulated with LPS, both IL-23 and IL-12 production was decreased (Fig B in S1 File). However, the TLR4 siRNA-dependent decrease of IL-12 production was much stronger than that of IL-23 production (Fig B in S1 File). These data suggest that IL-12 production in THP-1 cells is strongly dependent on TLR4, and IL-23 production is also related with TLR4.

*T*. *gondii*-induced IL-23 secretion was inhibited by both TLR2 siRNA transfection (81.4%, *P*<0.05) and TLR4 siRNA transfection (41.9%) compared to control siRNA transfection. However, *T*. *gondii*-induced IL-12 secretion was strongly inhibited by TLR4 siRNA transfection (87.9%, *P*<0.05) but slightly inhibited by TLR2 siRNA transfection (4.4%) compared to control siRNA transfection (Fig 3B). These results clearly demonstrate that *T*. *gondii*-induced IL-23 expression is mainly mediated via TLR2, whereas IL-12 expression is mainly dependent on TLR4.

### *T*. *gondii*-induced AKT phosphorylation depends on TLR2 and TLR4

**Fig 4:**
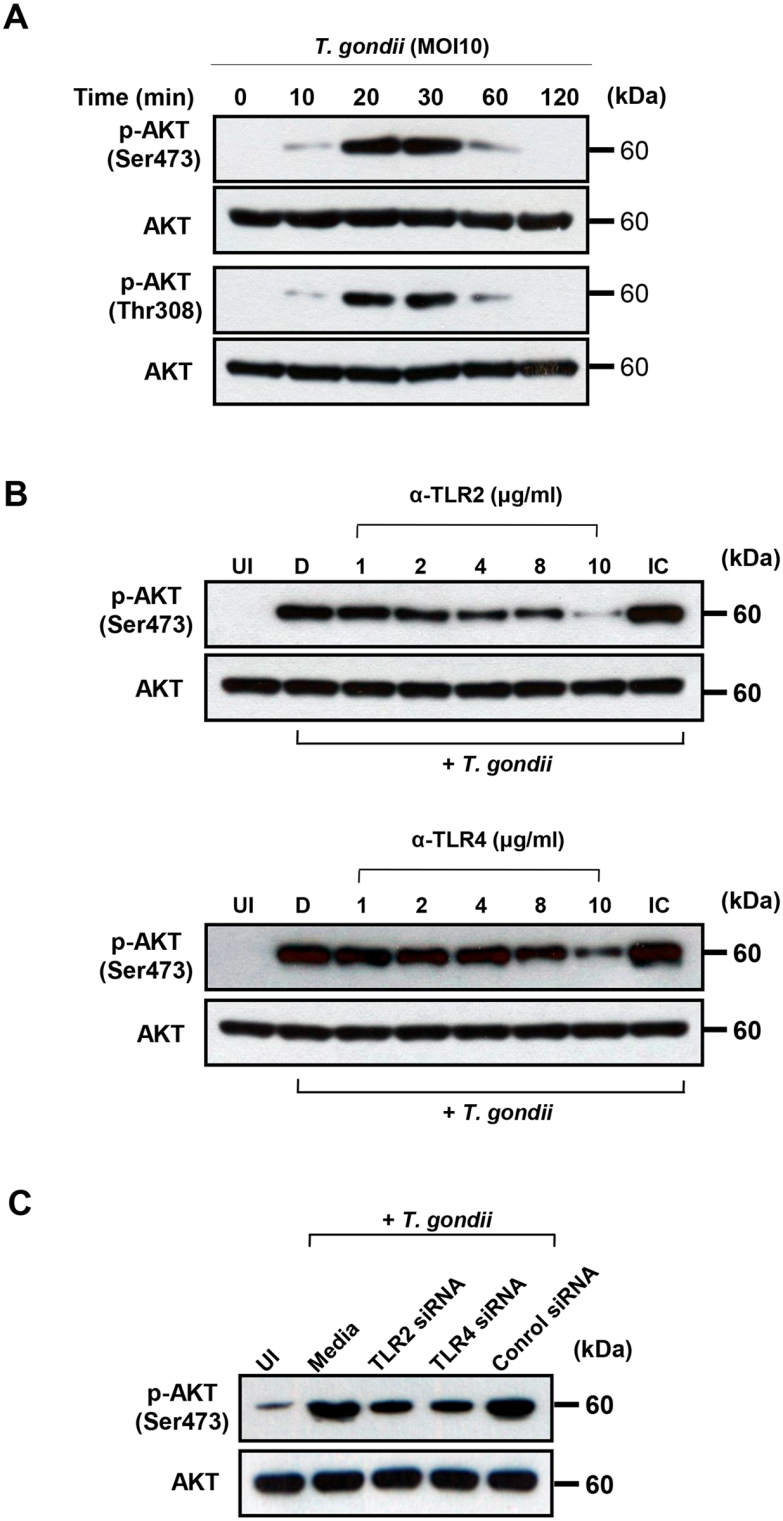
Regulation of AKT activation in *T*. *gondii*-infected THP-1 cells upon treatment with TLR2 or TLR4-specific antibodies or siRNA. (A) THP-1 cells were infected with *T*. *gondii* at MOI 10 for the indicated time points, cells were collected, and the levels of phospho-AKT (Ser473, Thr308) and total AKT were measured by western blot. (B) THP-1 cells were preincubated with the indicated amounts of anti-TLR2, anti-TLR4, or isotype-matched control (IC) 10 (μg/mL) and infected with *T*. *gondii* at MOI 10 for 30 min. Cells were collected, and the levels of phospho-AKT (Ser473) and total AKT were measured. (C) THP-1 cells were transfected with siRNA against TLR2 or TLR4 (or control siRNA) for 48 h and infected with *T*. *gondii* at MOI 10 for 30 min. Cells were collected, and the levels of phospho-AKT (Ser473) and total AKT were measured. A representative result of three independent replicates is shown.

To further determine the signaling pathways mediating the upregulation of IL-23 and IL-12 expression via TLR2 and TLR4, we first measured the activation of the PI3K-AKT pathway by *T*. *gondii* infection. The serine/threonine protein kinase B (PKB)/AKT mediates various downstream events of PI3K, and its activity is regulated by Thr308 and Ser473 phosphorylation [11]. *T*. *gondii*-induced phosphorylation of AKT at Ser473 and Thr308 occurred after 10 min post-infection, peaked after 20–30 min post-infection, and then decreased (Fig 4A).

Next, we examined how TLR2 or TLR4 signaling affects *T*. *gondii*-induced AKT activation. *T*. *gondii*-induced AKT phosphorylation was inhibited in THP-1 cells by pretreatment with anti-TLR2 mAb or anti-TLR4 mAb, but not isotype-matched control mAb, in a dose-dependent manner (Fig 4B). In addition, *T*. *gondii*-induced AKT phosphorylation was inhibited by transfection of siRNAs against TLR2 or TLR4 (Fig 4C), this results especially matches with IL-23 expression which was impacted both TLR2 and TLR4 knockdown (S1 File and Fig 3B). Taken together, these data demonstrate that *T*. *gondii*-induced AKT phosphorylation is mediated via both TLR2 and TLR4.

### Role of PI3K activity in modulating *T*. *gondii*-induced MAPK phosphorylation and critical roles in secretion of IL-23 and IL-12

**Fig 5:**
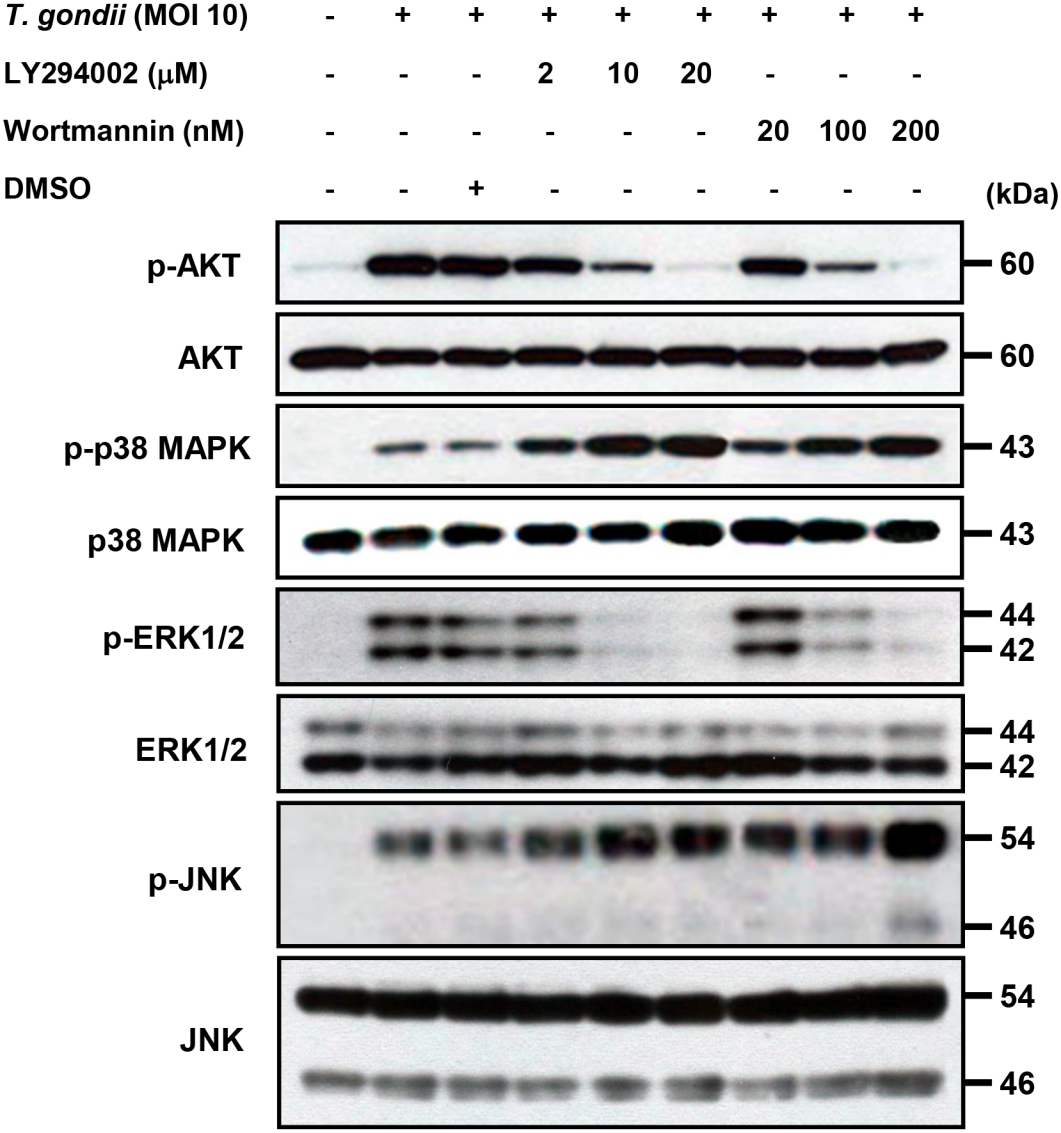
Regulation of AKT and MAPKs activations in *T*. *gondii*-infected THP-1 cells upon pretreatment with PI3K inhibitors. THP-1 monocytes were preincubated with the PI3K inhibitor LY294002 (LY) or wortmannin (WM) for 1 h, and then were infected with *T*. *gondii* at MOI 10 for 30 min. The cellular lysates were separated by SDS-PAGE and analyzed by immunoblotting using phospho-specific primary antibodies against AKT (Ser473), p38 MAPK, ERK1/2, and JNK. To ensure equal protein loading, the blots were stripped and reprobed with antibodies against AKT, p38 MAPK, ERK1/2, and JNK. A representative experiment of three independent replicates with similar results is shown.

The p38 MAPK, ERK1/2, JNK, and PI3K/AKT pathways have been reported to be involved in various cytokine productions and there is the possibility of crosstalk among these signaling pathways in the production of IL-12 and IL-23 in response to *T*. *gondii* infection. We pretreated cells with PI3K inhibitors before infection with *T*. *gondii*, and the activation of the MAPK pathways was determined by western blot analysis. *T*. *gondii* infection resulted in AKT phosphorylation, which was inhibited in a dose-dependent manner by pretreatment with LY294002 or wortmannin (Fig 5). In addition, pretreatment with 20 μM LY294002 or 200 nM wortmannin completely blocked *T*. *gondii*-induced AKT phosphorylation. *T*. *gondii*-induced phosphorylation of ERK1/2 was inhibited in a dose-dependent manner by pretreatment with LY294002 or wortmannin, suggesting that EKR1/2 activation is positively regulated by the PI3K pathway of *T*. *gondii*-infected THP-1 cells (Fig 5). In contrast, *T*. *gondii*-induced phosphorylation of p38 MAPK was markedly and phosphorylation of JNK was mildly enhanced by treatment with PI3K inhibitors, suggesting that the PI3K pathway negatively modulates *T*. *gondii*-induced activation of p38 MAPK and JNK (Fig 5). These data suggest that EKR1/2 activation in *T*. *gondii*-infected THP-1 cells is positively regulated by the PI3K pathway, whereas p38 MAPK and JNK activation negatively modulate this pathway.

**Fig 6:**
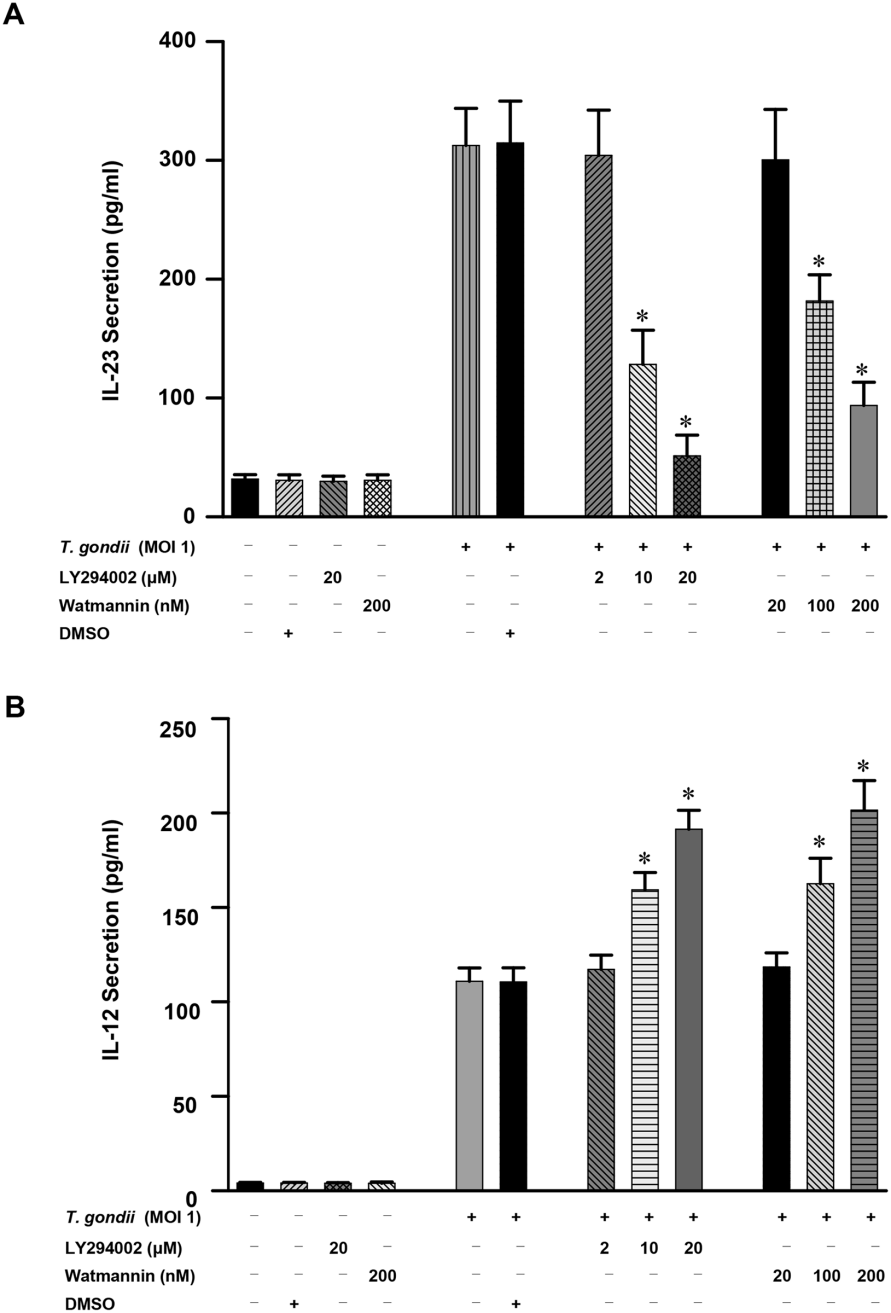
Differential regulation of IL-23 and IL-12 productions in *T*. *gondii*-infected THP-1 cells upon pretreatment with PI3K inhibitors. PI3K inhibitor LY294002 (LY) or wortmannin (WM) was added to THP-1 cells at the concentrations indicated at 1 h before infection with *T*. *gondii* at a MOI of 1. The supernatants were harvested after 18 h for cytokine assessment using ELISA. One representative experiment performed in triplicate is shown. The IL-23 (A) or IL-12 (B) levels (mean ± SD) following stimulation with *T*. *gondii*, as well as IL-23 and IL-12 production in the presence of the inhibitor, are shown. The solvent control was 0.1% DMSO. * *P*<0.05 compared with DMSO-treated *T*. *gondii*-infected group.

Next, we examined the role of PI3K-AKT signaling pathway in modulating *T*. *gondii*-induced production of IL-23 and IL-12 in human THP-1 cells. *T*. *gondii*-induced IL-23 production was dose-dependently inhibited by treatment with the PI3K inhibitors LY294002 and wortmannin. Pretreatment with 10 μM and 20 μM LY294002 significantly inhibited the production of IL-23 (*P*<0.05), and similar results were observed in cells pretreated with 100 nM and 200 nM wortmannin (*P*<0.05) (Fig 6A). This inhibition was not attributable to DMSO because the latter did not have any inhibitory effects at this concentration. In sharp contrast, *T*. *gondii*-induced IL-12 production was dose-dependently increased by treatment with the PI3K inhibitors LY294002 and wortmannin. Pretreatment with 10 μM and 20 μM LY294002 significantly increased the production of IL-12 (*P*<0.05), and similar results were also observed with 100 nM and 200 nM wortmannin pretreatment (*P*<0.05) (Fig 6B). These data indicate that the PI3K signaling pathways play a positive role in the *T*. *gondii*-induced secretion of IL-23 secretion but a negative role in *T*. *gondii*-induced IL-12 secretion.

### MAPK pathways have different roles in *T*. *gondii*-induced IL-23 and IL-12 production

**Fig 7:**
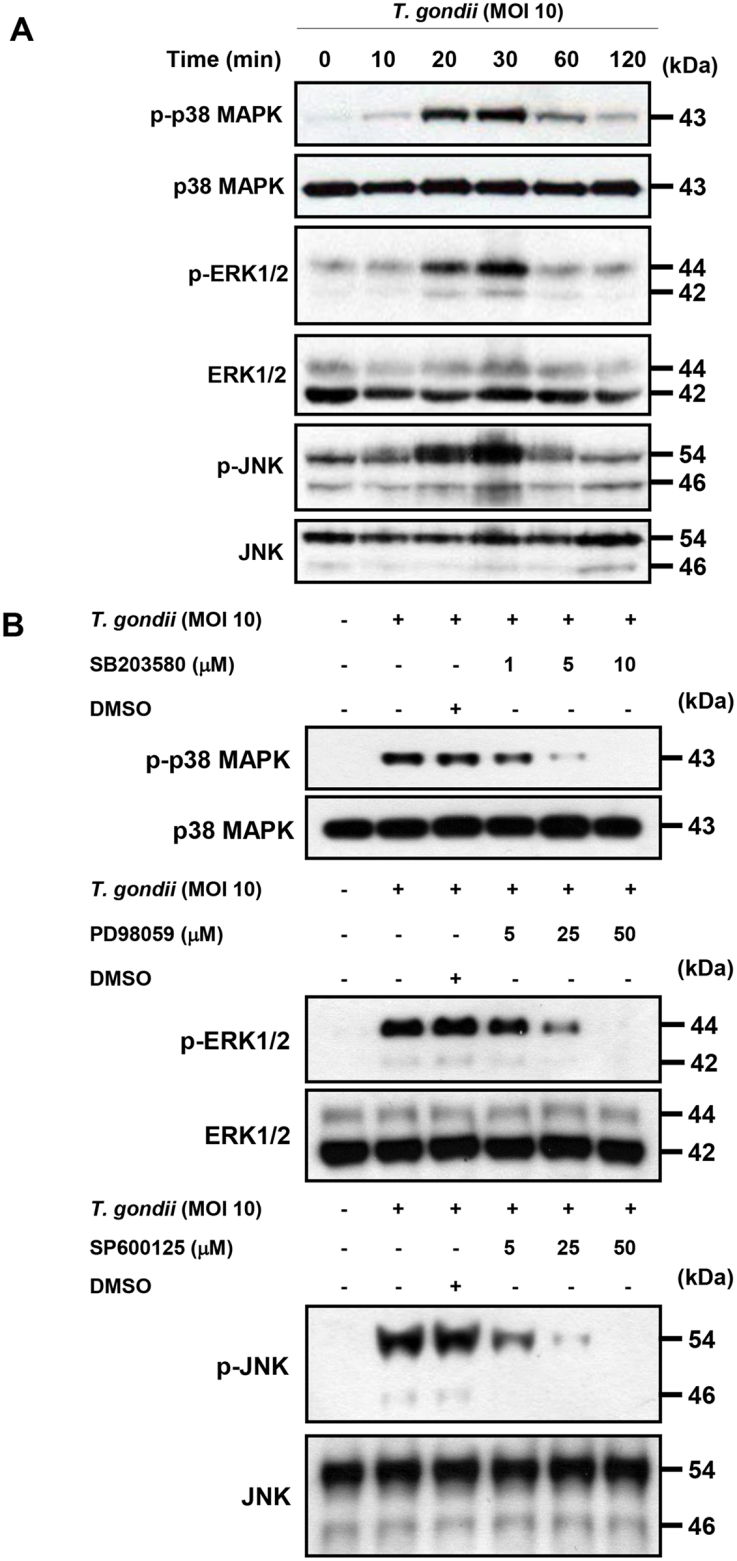
Regulation of p38 MAPK, ERK1/2 or JNK activation in *T*. *gondii*-infected THP-1 cells by specific inhibitors. (A)THP-1 cells were infected with *T*. *gondii* at MOI 10 for the indicated time and the phosphorylation levels of p38 MAPK, ERK1/2 and JNK was checked by western blot. (B) THP-1 cells were pretreated with the p38 inhibitor (SB203580), ERK inhibitor (PD98059), or JNK inhibitor (SP600125) for the indicated concentration for 1 h and infected with *T*. *gondii* at MOI 10 for 30 min. The phosphorylation levels of p38 MAPK, ERK1/2 and JNK was checked. A representative experiment of three independent replicates with similar results is shown.

THP-1 cells exposed to live *T*. *gondii* tachyzoites underwent rapid activation ofp38 MAPK, ERK1/2, and JNK (Fig 7A). Pretreatment with p38 MAPK (SB203580), ERK1/2 (PD98059) or JNK (SP600125) inhibitors inhibited *T*. *gondii*-induced phosphorylation of p38 MAPK, ERK1/2 or JNK in a dose-dependent manner, respectively. Furthermore, *T*. *gondii*-induced p38, ERK1/2 or JNK phosphorylation were completely inhibited by pretreatment with 10 M SB203580, 50 M PD98059 or 50 M SP600125 inhibitors respectively (Fig 7B). However, phosphorylation levels of the other MAPK subtypes were not changed significantly after treatment with specific inhibitors according to MAPK subtypes (the data were not shown). These results clearly demonstrated that manipulation of the AKT and MAPK signaling pathways with tested specific inhibitors is effective in our in vitro experimental model.

**Fig 8:**
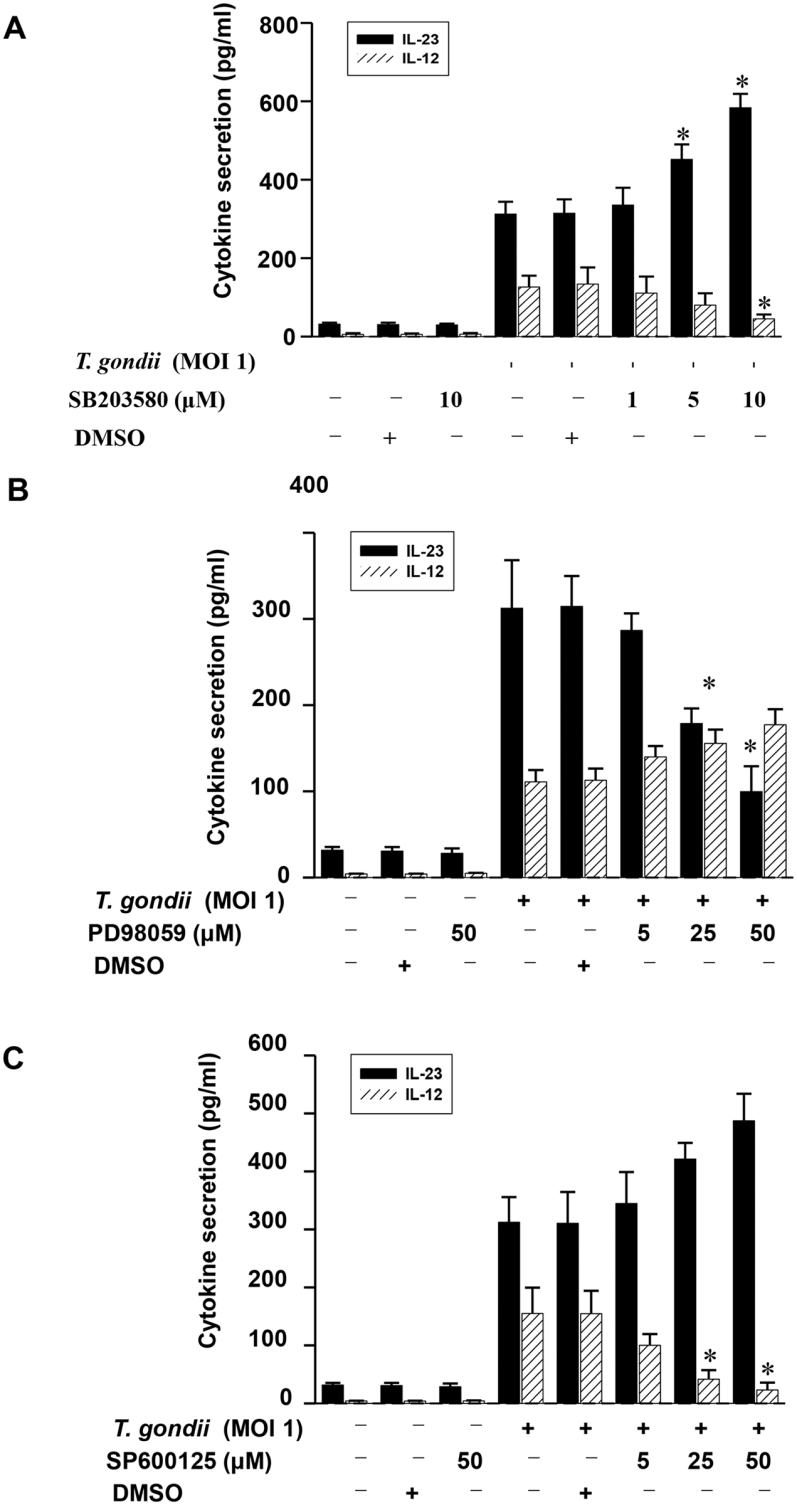
Differential regulation of IL-23 and IL-12 productions in *T*. *gondii*-infected THP-1 cells upon pretreatment with specific inhibitor of p38 MAPK, ERK1/2 or JNK. The p38 inhibitor (SB203580), ERK inhibitor (PD98059), or JNK inhibitor (SP600125) was added to THP-1 cells at the concentrations indicated at 1 h before infection with *T*. *gondii*. The culture supernatants were harvested after 18 h for cytokine assessment using ELISA. The IL-23 and IL-12 levels following stimulation with *T*. *gondii* with or without the presence of p38 MAPK inhibitor (A), ERK1/2 inhibitor (B), or JNK inhibitor (C) are shown. The solvent control was 0.1% DMSO. One representative experiment performed in triplicate is shown.* *P*<0.05 compared with DMSO-treated *T*. *gondii*-infected group.

To further determine the specific involvement of each MAPK pathway in the regulation of IL-23 and IL-12 production, we employed the inhibitors corresponding to each signal pathway. IL-23 production in *T*. *gondii*-infected THP-1 cells was significantly upregulated by the p38 MAPK inhibitor SB203580 in a dose-dependent manner, whereas *T*. *gondii*-stimulated IL-12 production was significantly downregulated by treatment with 10 μM SB203580 (Fig 8A). The inhibition of ERK1/2 before stimulation with *T*. *gondii* resulted insignificant downregulation of IL-23 production. However, pretreatment with PD98059 had no effect on *T*. *gondii*-stimulated IL-12 production (Fig 8B). Inhibition of the JNK pathway with SP600125 had no effect on *T*. *gondii*-induced IL-23 production but significantly downregulated IL-12 production in a dose-dependent manner (Fig 8C). In summary, these results clearly indicate that *T*. *gondii*-induced p38 MAPK phosphorylation negatively regulates IL-23 production and positively regulates IL-12 production. However, *T*. *gondii*-induced ERK1/2 and JNK phosphorylation positively regulate IL-23 and IL-12 production, respectively.

**Fig 9:**
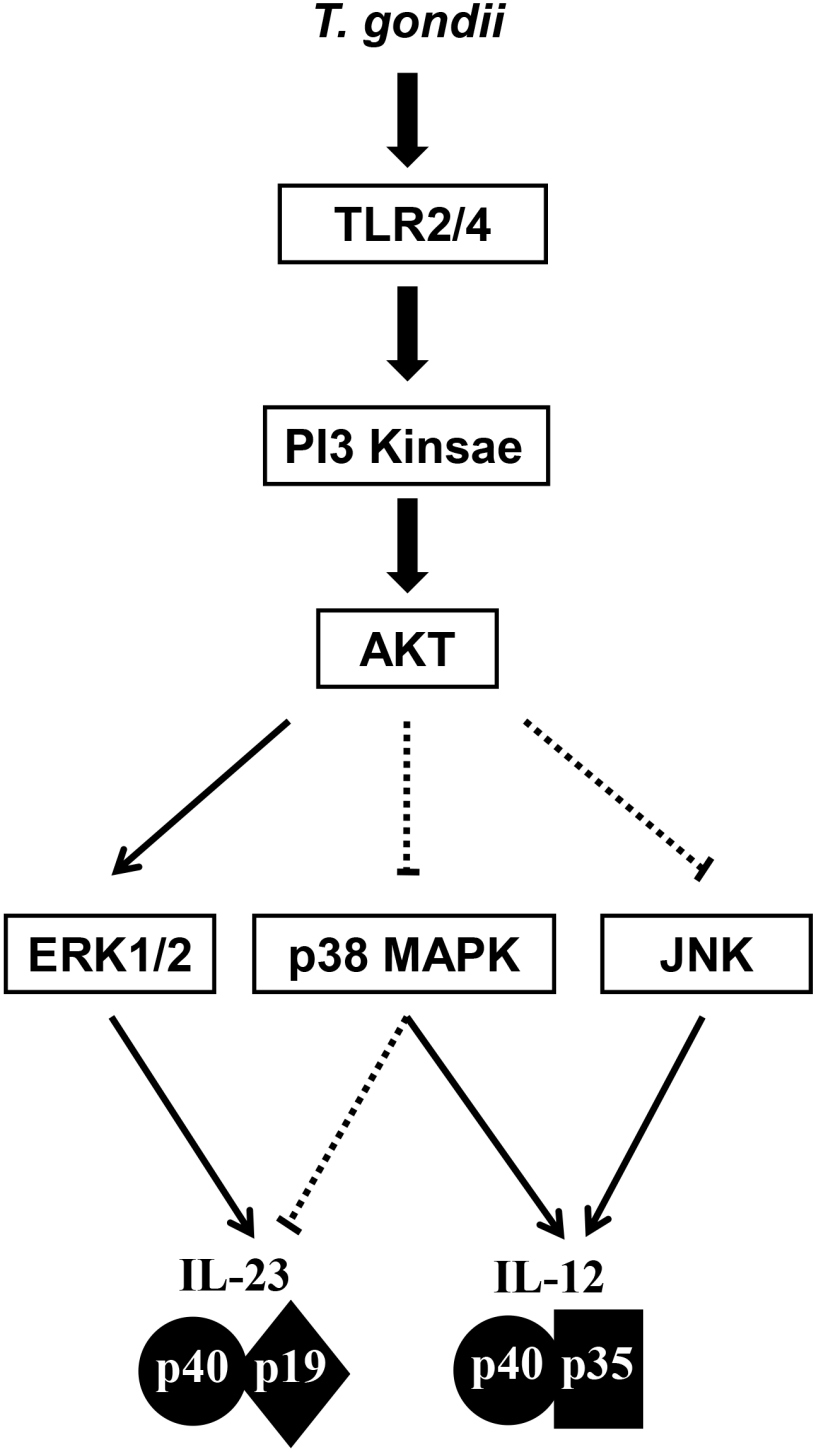
Hypothetical networks of PI3K/AKT and MAPK signaling pathwaysinvolved in the differential regulation of *T*. *gondii*-induced IL-23 and IL-12 production in THP-1 cells. The PI3K/AKT and MAPK pathways are activated by *T*. *gondii*-infection via TLR2 and TLR4. AKT activation differentially modulates ERK1/2, p38 MAPK, and JNK activation; PI3K/AKT inhibition suppresses ERK activation, whereas inhibition of PI3K/AKT results in the induction of p38 MAPK and JNK. Bold lines indicate a positive regulation pathway, and dotted lines indicate the negative regulation pathway of IL-23 and IL-12 production.

In sum, IL-23 and IL-12 production in *T*. *gondii*-infected THP-1 cells was mainly mediated via TLR2 and TLR4, respectively. IL-23 production is positively regulated by PI3K and ERK1/2 but negatively regulated by p38 MAPK. In sharp contrast, IL-12 production is negatively regulated by PI3K and positively regulated by p38 MAPK and JNK (Fig 9).

## Discussion

IL-23 and IL-12 are important cytokines to bridge innate and adaptive immunity, and work in concert to regulate the cellular immune responses critical for the host defense. However, their differential roles of IL-12 and IL-23 were not much addressed in the immune responses during *T*. *gondii* infection. Previously, we reported that IL-23 secretion was significantly higher than IL-12 secretion at THP-1 human monocytic cells in response to either live tachyzoites, excretory/secretory proteins or soluble antigen of *T*. *gondii* RH strain even though the secretion patterns of IL-23 and IL-12 were similar [18], suggesting the possible some different signaling pathways in production of IL-23 and IL-12 in *T*. *gondii*-infected monocytes. Here, we investigated the intracellular cross-talks among TLR2/4, PI3K/AKT and MAPK signaling in the regulation of IL-12 and IL-23 production by *T*. *gondii*-infected THP-1cells. We found that IL-23 and IL-12 production in *T*. *gondii*-infected THP-1 cells was mainly mediated via TLR2 and TLR4, respectively. In THP-1 cells infected with *T*. *gondii* RH strain, IL-23 production is positively regulated by PI3K and ERK1/2 but negatively regulated by p38 MAPK. In sharp contrast, IL-12 production is negatively regulated by PI3K and positively regulated by p38 MAPK and JNK (Fig 9).

Inflammatory monocytes have been appreciated as important in mucosal immunity against the parasite *T*. *gondii*, and likely other enteric pathogens [19–21]. Monocyte recruitment relies in induction of MCP-1 and requires the chemokine receptor CCR2; in their absence mice rapidly succumbed to nonlethal challenge with a normally avirulent strain of *T*. *gondii* [19], and the inflammatory monocytes revealed the critical role during the acute infection with *T*. *gondii* and neutrophils are not protective but rather contribute to the pathology [20]. In the oral animal model for toxoplasmosis, monocytes elicited by *T*. *gondii* infection are equipped with several effector mechanisms, including the production of IL-12, secretion of TNF-α, upregulation of MHC class II, and induction of iNOS expression [21]. Therefore, it is worth to elucidate the regulation mechanism for differential IL-12 and IL-23 production in monocytes during *T*. *gondii* infection, since the role of monocytes get critical in the initial phase of *T*. *gondii* infection. In our studies, we addressed the specific signaling mechanisms for differential regulation of IL-12 and IL-23 production in an encounter between monocytes and *T*. *gondii*, even though our experimental model may not appreciate all the aspects relevant to the interaction between *T*. *gondii* and monocytes in vivo. The primary infection has been carried out in vitro using exclusively tachyzoites, using a single human monocyte cell line without any other cellular interactions. The present study provides new understanding of the specific role of the PI3K/AKT and MAPK pathways for the differential production of *T*. *gondii*-induced IL-23 and IL-12. In general, it is important to understand the signaling mechanism for the proper balance between IL-23 and IL-12, because it plays a key immunoregulatory role in the progress of pathological Th1-mediated autoimmune and inflammatory disease [22].

There were many reports about the IL-12 production in *T*. *gondii*-infected cells [17,23]. IL-12 induces protective responses against *T*. *gondii*, including CD8^+^ T cell cytotoxic and IFN-γ responses from Th1 and NK cells [23]. However, not much is known about the role and production of IL-23 during *T*. *gondii* infection.IL-23 was reported to mediate *T*. *gondii*-induced immunopathology in inflammation in the small intestine via matrix metalloproteinase-2 and IL-22, independent of IL-17 [24]. In the absence of IL-12, IL-23 has protective effects against *T*. *gondii* infection, although IL-12 plays a dominant role in resistance to toxoplasmosis [25]. Thus, IL-23 is key mediator of autoimmune inflammation but may play a limited protective role against an intracellular pathogen, whereas IL-12 is indispensable for intracellular parasitic infections such as toxoplasmosis [4]. These data suggest that IL-23 and IL-12 play separate but compensatory roles during innate immunity and also their productions are regulated through different signaling pathways.

TLRs are important in host resistance, and there is evidence that TLR2, 4, 9 and 11 are involved in recognition of *T*. *gondii* although knockout of no single TLR results in the high susceptibility observed following infection of MyD88^−/−^ mice [10,26,27]. Regarding the involvement of TLR2 and TLR4 in *T*. *gondii* infection, core glycans and lipid moieties obtained from glycosylphosphatidylinositol-anchored proteins on the tachyzoite surface were recognized by both TLR2 and TLR4 [10]. In the present study, we found the differential roles of TLR2 and TLR4 signaling pathways in IL-23 and IL-12 production in *T*. *gondii*-infected THP-1 cells. The TLR2 signaling pathway played a major role for the *T*. *gondii*-induced IL-23 production in THP-1 cells. The IL-12 production was regulated by the TLR4 signaling pathway, not by the TLR2 signaling way. Importantly, TLR2-deficient mice were shown to be susceptible to infection with very high doses of *T*. *gondii*, although they mount an unimpaired immune response when infected with conventional parasite loads [28]. In addition, infection with high doses of *Mycobacterium tuberculosis* resulted in rapid mortality in TLR2-deficient mice [29]. TLR2 regulated tumor necrosis factor (TNF) and CC-chemokine ligand 2 (CCL2) productions by macrophages and neutrophils, respectively, although TLR2 deficiency did not have a major effect on IL-12 production during *T*. *gondii* infection [30]. Our data provide insights into the role of TLR2 in infection with *T*. *gondii*, with implications for the pathophysiology of the disease. Further studies are required to understand the precise biological function of TLR2 in terms of cytokine production in monocytes in response to *T*. *gondii* infection.

Next, we investigated PI3K/AKT signaling pathway as a TLR2/TLR4 downstream pathway involved in the production of IL-23 and IL-12 in *T*. *gondii*-infected THP-1 cells. In this study, we found the differential role of PI3K/AKT pathway in the production of IL-23 and IL-12 in *T*. *gondii*-infected THP-1 cells. AKT phosphorylation was increased soon after *T*. *gondii* infection in THP-1 cells, which was consistent with another report [12]. More, *T*. *gondii*-induced AKT phosphorylation was inhibited by treatment of anti-TLR2 or anti-TLR4 antibodies, suggesting the PI3K/AKT pathway are downstream of TLR2/4 signaling. Interestingly, IL-23 production in response to *T*. *gondii* infection was positively regulated by PI3K, whereas IL-12 production was negatively regulated. Similar to our results, it was reported that PI3K negatively regulates IL-12 synthesis by DCs with *Leishmania major* infection [31], and PI3K inhibitors significantly decrease IL-23 expression in chronic viral hepatitis [32]. These results may correspond to the idea that TLRs initiate a proinflammatory response via a positive feedback loop after microorganism infection, while activating PI3K as a compensatory negative feedback pathway to limit excessive inflammatory signaling [31,33].

MAPK pathways have been known to be important regulators in proinflammatory cytokine production [34]. However, a clear consensus has not been reached concerning their roles in the regulation of *T*. *gondii*-induced IL-23 production. We observed that *T*. *gondii*-infected THP-1 cells resulted in rapid activation of ERK1/2, JNK, and p38 MAPK, consistent with previous results [14]. Importantly, we found that *T*. *gondii*-induced p38 MAPK phosphorylation negatively regulated IL-23 production and positively regulated IL-12 production. These results are consistent with previous data that IL-12 production in macrophages in response to *T*. *gondii* is dependent on the phosphorylation of p38 MAPK through TRAF6 [15] or the TAB1-dependent p38 autophosphorylation [35]. We also found that *T*. *gondii*-induced ERK1/2 and JNK phosphorylation positively regulates IL-23 and IL-12 production, respectively; these results were similar to previously demonstrated that ERK1/2 activation is required for HBV viral protein HBx-induced IL-23 expression in hepatitis B virus (HBV) infection [32].

Taken together, production of IL-12 and IL-23 in the *T*. *gondii*-infected human THP-1 monocytic cells was differentially regulated by the signaling networks of the PI3K and MAPK pathways downstream of TLR2/4 signaling. Our results indicate that IL-23 production was regulated mainly by TLR2 and then by PI3K and ERK1/2; however, IL-12 production was mainly regulated by TLR4 and then by p38 MAPK and JNK. In *T*. *gondii*-infected THP-1 cells, there are some cross-talks between PI3K pathway and MAPK activation; ERK1/2 activation was activated by PI3K; however, the phosphorylation of p38 MAPK and JNK was negatively modulated by the PI3K signaling pathway.

## Supporting Information

S1 Fig Viability of THP-1 cells at 18 h after *T*. *gondii* infection. The percentage of the live THP-1 cells treated with *T*. *gondii* at a MOI of 0, 0.1, 1, or 10 for 18 h were assayed by using CellTiter 96^®^AQueous One Solution Cell Proliferation Assay Kit (Promega, Madison, WI. USA).
(TIF)

S1 File IL-23 and IL-12 production levels in THP-1 cells transfected with siRNAs against TLR2 or TLR4 (or control siRNA) after incubation with TLR2 and TLR4 agonist. siRNAs against TLR2 or TLR4 (or control siRNA) transfected THP-1 cells were incubated with Pam_3_CSK_4_ (TLR2 agonist) or LPS (TLR4 agonists) for 18 h. Production of IL-23 and IL-12 was measured by ELISA in the supernatants of THP-1 cells treated with control siRNA or siRNA against TLR2 (siTLR2) or TLR4 (siTLR4) and subsequently with Pam_3_CSK_4_ (Fig A) or LPS (Fig B), respectively. * *P*<0.05 compared with control siRNA transfected THP-1 cells treated with each indicated concentration of Pam_3_CSK_4_ or LPS.
(TIF)
