# Supplementary figures and images for: Intracellular Networks of the PI3K/AKT and MAPK Pathways for Regulating Toxoplasma gondii-Induced IL-23 and IL-12 Production in Human THP-1 Cells
Source: PLoS One. 2015 Nov 3;10(11):e0141550. doi: 10.1371/journal.pone.0141550 (PMC4631599; doi:10.1371/journal.pone.0141550)

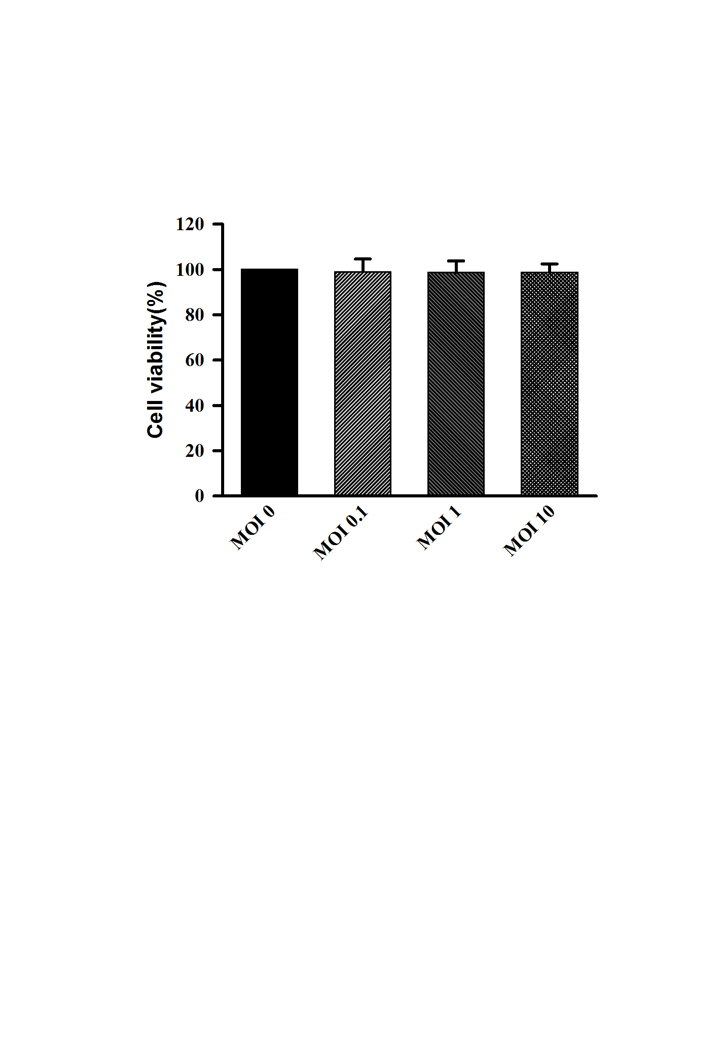

Supplement: S1 Fig — The percentage of the live THP-1 cells treated with T. gondii at a MOI of 0, 0.1, 1, or 10 for 18 h were assayed by using CellTiter 96®AQueous One Solution Cell Proliferation Assay Kit (Promega, Madison, WI. USA). (TIF) [file pone.0141550.s001.tif]

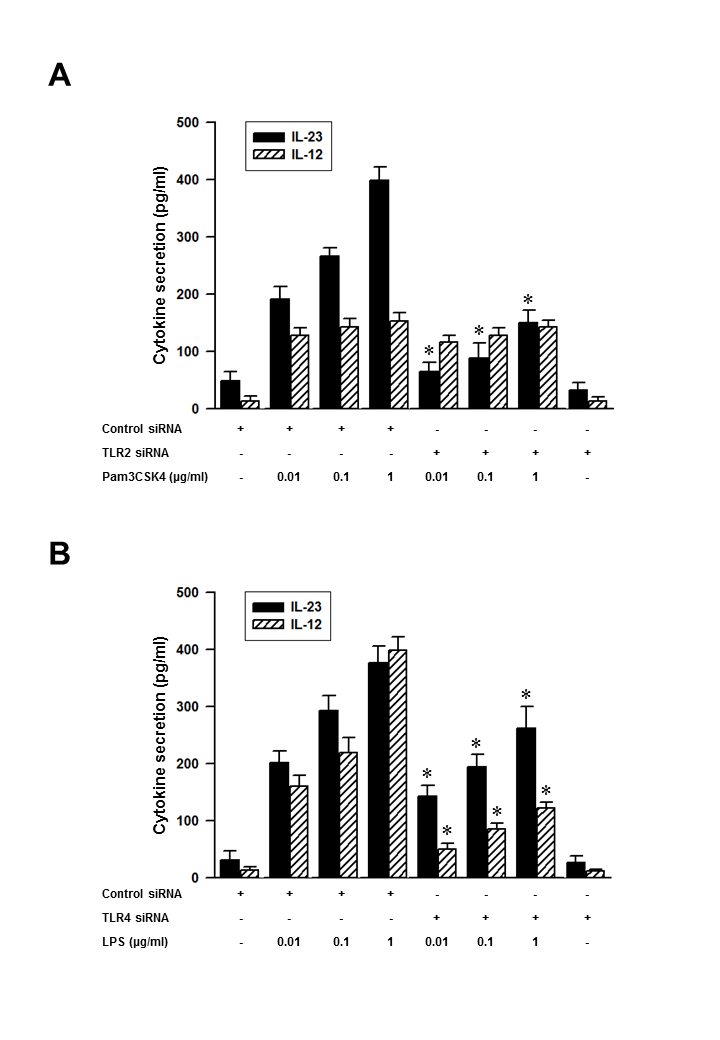

Supplement: S1 File — siRNAs against TLR2 or TLR4 (or control siRNA) transfected THP-1 cells were incubated with Pam3CSK4 (TLR2 agonist) or LPS (TLR4 agonists) for 18 h. Production of IL-23 and IL-12 was measured by ELISA in the supernatants of THP-1 cells treated with control siRNA or siRNA against TLR2 (siTLR2) or TLR4 (siTLR4) and subsequently with Pam3CSK4 (Fig A) or LPS (Fig B), respectively. * P<0.05 compared with control siRNA transfected THP-1 cells treated with each indicated concentration of Pam3CSK4 or LPS. (TIF) [file pone.0141550.s002.tif]
